# Supplementary material for: Intelligibility improves perception of timing changes in speech
Source: PLoS One. 2023 Jan 12;18(1):e0279024. doi: 10.1371/journal.pone.0279024 (PMC9836318; doi:10.1371/journal.pone.0279024)
Supplement: Attachment — Submitted filename: shift_detection_REBUTTAL_FINAL.pdf [file pone.0279024.s001.pdf]

Editor

Both reviewers had positive things to say about the manuscript, but I agree that greater clarity in definitions and some further explication around the rationale and interpretations would be useful. In addition, further support for some experimental interpretations (either experimental support or further support from existing literature) would be helpful.

We thank the Editor and both Reviewers for their helpful comments that allowed us to improve our submission. We revised our manuscript according to these comments. In particular, we included additional analyses and clarifications suggested by Reviewer 1, added a more precise definition of our aims and rationale to address Reviewer 2's concerns, and clarified how we think our results need to be interpreted. We provide point-by-point responses below. We have also addressed additional requirements listed in the decision email (including Data Availability Statement).

Reviewer #1:

GENERAL REMARKS

In this manuscript, the authors investigate whether or not intelligibility impacts listeners' ability to detect rhythmic irregularities in speech. They report the results of three experiments. In the first experiment, they replicate their previous finding that irregularity detection is better for intelligible (16-channel noise-vocoded) compared to unintelligible (1-channel noise-vocoded) speech. In the second experiment, they rule out the possibility that this result was due to enhanced spectral complexity by demonstrating better irregularity detection for intelligible 16-channel noise-vocoded speech compared to largely unintelligible 16-channel spectrally-rotated noise-vocoded speech. In the third experiment, they attempt to remove any possible acoustic confounds by using sine-wave speech stimuli and comparing irregularity detection when listeners were trained or untrained in perceiving the stimuli as speech. Results again support the idea that intelligibility aids irregularity detection.

I very much enjoyed reading the paper: it is clear, well-written and compelling. The results are thoughtfully discussed in the light of existing findings/theories and plausible hypotheses are generated about potential underlying mechanisms. The authors also highlight some possible implications for motor-based theories of speech perception. Additionally, and as a proponent

of online testing, I was heartened to see replication of results obtained in the lab via an internet-based implementation.

My comments are mostly very minor requests for clarification or additional information.

We are glad the Reviewer enjoyed reading our manuscript and are grateful for positive comments and additional suggestions. We have revised our manuscript accordingly.

MAJOR COMMENTS

1. Carry-over effects. If I've understood correctly, then the stimuli were always presented at the same rate. If that's the case, I wonder whether there were "carry-over effects" from trial to trial. In other words, did listeners gradually build up increasingly strong expectations about speech rate, thus increasing their sensitivity to irregularities over the course of each experiment? Or was there some kind of perceptual "reset" between items, meaning that listeners started each trial with a blank slate, temporally-speaking? N.B. This is different to the "practice effect" identified in Exp 3: in that case, the issue was whether listeners had started to hear the stimuli as speech through mere exposure; in this case, the build-up of temporal expectations could function independently of intelligibility. Having said that, though, I could easily imagine that intelligibility might differentially affect performance for early vs. late trials if there is indeed a build-up of temporal expectations over time. I appreciate the need to pool data for the sake of the SDT-based statistical analyses, but it would be interesting to see if sensitivity changed over time for the intelligible vs. unintelligible conditions of Exps 1 and 2 (by comparing e.g. early vs. late trials).

We thank the Reviewer for this interesting suggestion. We have included an investigation into carry-over effects, focusing on Experiment 2 due to the higher number of subjects. We split the experiment into quartiles and extracted for each quartile the average sensitivity (d-prime) in the irregularity detection task. We did not find evidence for systematic changes in sensitivity over time, as shown in the figure below (shaded area shows SEM). We now report this result in our manuscript (p. 21 – all page numbers refer to the version with tracked changes):

"We also tested whether sensitivity in the irregularity detection task, as well as differences across conditions, changed over the course of the experiment. To do so, we divided trials into quartiles according to their timing during the experiment, and compared performance between those quartiles. We did not find a main effect of quartile on d-prime (F(3) = 0.44, p = 0.73), nor an interaction between quartile and condition (F(6) = 0.71, p = 0.64). This indicates that performance was relatively stable throughout the experiment."

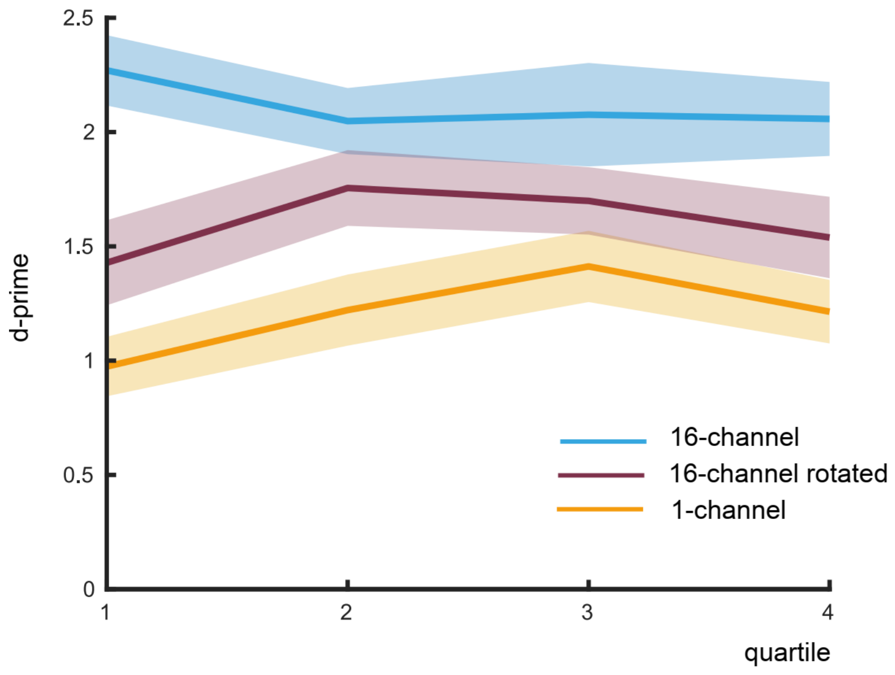

2. Experiment 3 stimuli. Thanks to the authors for making example stimuli available via the OSF! Having listened to these, I have to say that the sine-wave speech stimuli used in Exp 3 already sound very speech-like (and indeed intelligible) to me (although I do have experience working with SWS). More to the point, most participants already seem to be identifying the SWS as speech from very early on (Fig. 5A). I appreciate that the effect of interest in Exp 3 is actually the training/practice effect, which is clearly contributing to improved irregularity detection. However, I wonder if the authors could be clearer that this improved sensitivity is due to an *enhancement* of (a likely already existing) perception of speech/intelligibility rather than a binary distinction between perceiving the stimuli as "speech" or "not speech" (this binary distinction being strongly implied by some phrasing e.g. p38, line 784). In other words, there's a continuum when it comes to the perception of speech-like-ness/intelligibility in the stimuli, and a shift along this continuum seems to affect the perception of rhythmic regularity.

We agree with the Reviewer and have now made it clearer that training enhanced participants' perception of sine-wave speech rather than changing it categorically:

Results (p. 30):
"Although a relatively large proportion of participants spontaneously described the stimulus as speech, this proportion increased after training by 22.0 %, 32.2 %, and 57.5 % in Groups 1, 2, and 3, respectively (based on responses immediately after vs immediately before training, see Procedure)."

Discussion (p. 34):
"In Experiment 3, we eliminated any acoustic differences between conditions and instead manipulated participants' perception of a single set of sine-wave speech stimuli, by training groups of participants to perceive the stimulus as speech at different points throughout the experiment. Given the relatively large proportion of participants that spontaneously identified this stimulus as speech, it is likely that the training enhanced speech perception rather than changing it categorically. Nonetheless, the effect of training was to substantially modify speech intelligibility without changing the acoustic form of our sine-wave speech stimuli – which achieves our empirical goals."

3. Experiment 3 sample. I understand the authors' motivation for recruiting a smaller sample size for Group 3 (lines 568-569), but is there a reason for the specific sample size chosen? It would be good if the authors could be explicit about this (even if it was dictated by time/finances etc.!).

We are now more explicit about the sample size for Group 3 (p. 25):
"As these two groups (totalling 119 participants) provided us with a very reliable estimate of speech rhythm perception in naïve participants (in their first experimental block), we halved the sample size for Group 3 […]."

4. Experiment 3 training. It seems a little misleading to describe the training as "successful" on the basis that more participants described the stimuli as speech, given that the training explicitly tells participants that the stimuli were derived from speech! (p34, lines 683-4.) Would it not be better to examine the relationship between whether or not participants described the stimuli as

speech and how they performed on the word reporting task? I also wonder about individual differences: my experience with SWS training is that it's rather binary – some participants learn it almost immediately and others never quite get it. Could the authors comment on individual differences in their data in terms of whether and to what extent training and/or practice led to changes in irregularity detection?

We agree with the Reviewer and removed the term "successful" (and the corresponding sentence).
We refrained from correlating participants' perception of sine-wave speech and performance in the word report task, as the latter was measured after the experiment, when almost all participants (>93 % in all three groups) described the stimulus as "speech".

We thank the Reviewer for the interesting suggestion to look at individual differences in practice and training effects. The binary effect (some participants learn and others do not) would predict a bimodal distribution which, however, we did not observe. Below we show histograms for practice and training effects that correspond to results shown in Figure 6B.

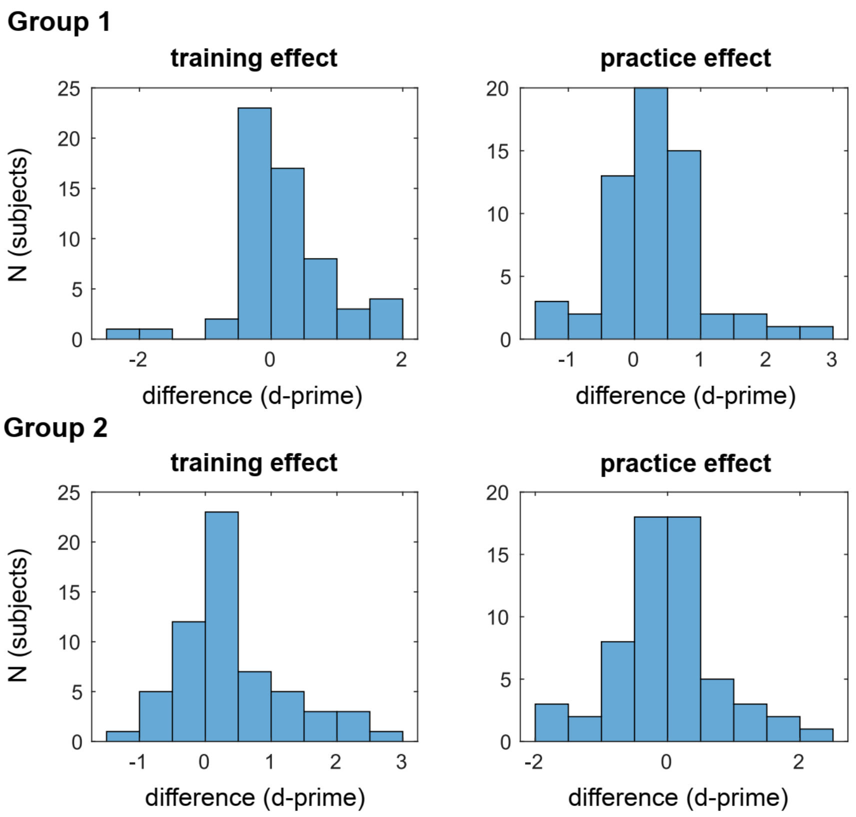

5. Experiments 2 and 3 online recruitment and procedures. a) The authors state that participants were recruited via Prolific and were "native speakers of English". I'm curious as to why other Prolific filters were not used. In particular, I wonder whether the authors considered restricting the sample to monolingual English speakers (given potential differences in speech rhythm perception across languages)

We appreciate the Reviewer's suggestion and included in the manuscript (p. 36/37):
"In the current study, all native speakers of English were eligible to participate, irrespective of their proficiency in other languages (e.g., bilingualism) or other factors. It would be interesting to test whether differences in rhythmic patterns between languages (e.g., stress-vs syllable-timed [4]) can influence results reported here."

[4] Allen GD. Speech Rhythm: Its Relation to Performance Universals and Articulatory Timing. J Phon. 1975.

and/or restricting the age range (given potential age-related changes in speech perception abilities).
Prolific also has a filter for self-reported hearing loss or hearing difficulties, which may have been worth applying here.

As now described on p. 14, *"we did not screen for age-related hearing difficulties or other conditions that can compromise speech perception abilities, as these are unlikely to affect the relative difference between (e.g., intelligible and unintelligible) conditions. However, we did make sure participants could pass a basic hearing test (with their volume set at a comfortable level), were wearing headphones (in a test described below) and could complete the task (based on performance in practice trials, described below)."*

b) I appreciate that the data from the pseudo-audiogram procedure (described on p21, lines 355-361) were collected for a different experiment, but might it be possible to report the data here nevertheless, to give the reader some indication of the participants' hearing sensitivity (especially given the extended age range, as already mentioned)?

We have included the following results:
For Experiment 2 (p. 17), *"on average, participants' hearing range measures were 58.64 dB (SD: 10.93 dB) below their self-adjusted comfortable volume level."*
For Experiment 3 (p. 26), *"similar to Experiment 2, participants' hearing range was estimated as 66.19 dB +/- 11.84 dB (mean +/-SD) below their self-adjusted comfortable volume level."*

c) What was the "device check" (p20, line 331) that detected use of phones/tablets?

We apologize for not clarifying this. The device check was some JavaScript code that checks for mobile devices using the browser's 'user agent string', which contains information about the device and browser that the participant is using to access the webpage. This check was not perceptible to participants, and would either produce an error page (if a mobile device was detected) or silently move on with the experiment. We have now clarified this in the Procedure (p. 15):
"Mobile phones and tablets were ineligible, which was ensured with a JavaScript-based device check that ran silently at the start of the study."

6. Experiment 1 procedure. What was the presentation level of the sounds and what device was used for presentation?

We have now clarified this in the Experiment 1 Procedure, p. 10: *"Participants used Sennheiser HD 202 headphones to listen to the rhythmic sequences, delivered by a standard sound card at an individually comfortable level."*

7. One final and very minor comment: in all three experiments, rhythmic irregularity was created by varying the position of the second, third or fourth word (W2, W3, W4) in a five-word sequence. However, it seems to me that varying the position of the second word is effectively the same as varying the position of the third word: if listeners assume the first IOI (i.e. the gap between W1 and W2) to be the "true" IOI, then it is the onset of W3 in these "W2-shift" stimuli which creates the percept of irregularity. The upshot of this is that there are effectively twice as many stimuli where attending to W3 (as opposed to W4) could enhance irregularity detection. A related, but slightly different, point is that stimuli where the irregularity occurs on W3 or W4 entail a phase (but not period) reset, whereas stimuli where the irregularity

occurs on W2 could be thought of as entailing both a phase and period reset. Of course, both of these things would be the case for all presented conditions, and therefore shouldn't impact between-condition comparisons – plus this comment only holds true if there's no gradual build-up of temporal expectations throughout the course of the experiment (see my first point above). Basically, I'm just curious to know if the authors have thought about this at all!

We thank the Reviewer for this interesting comment and included in our revised manuscript (p. 10/11):
"Note that, although irregularities were introduced pseudo-randomly, some might have been more salient than others: For instance, for irregular second syllables, only the occurrence of the third syllable creates the percept of irregularity. These differences, however, were present for all conditions and cannot affect comparisons between them."

MINOR COMMENTS, TYPOS ETC.

p13, line 147: participant's => participants'
p17, line 260: control of Type II => control Type II
p24, lines 427-434: check direction of quotation marks in parenthesised scales
p24, line 435: participants' => participants
p28, line 524: performance the block => performance in the block
p29, line 564: those experiment => those experiments
p41, line 849: amplitude modulations peaks => amplitude modulation peaks
p43, line 905: perception can only => perception which can only

Thank you, these typos have been corrected.

Many thanks to the authors for a stimulating and enjoyable paper!
Many thanks for reading our paper and for these comments which helped us improve it.

Reviewer #2:

Summary

The manuscript reports the results of three experiments conducted with spectrally degraded acoustic signals resembling real speech to different degrees. The goal of the experiments is to demonstrate that the perception of certain timing regularities in the presentation of isolated words is only accurate when the acoustic signals are interpretable as spoken language. I think the studies and their results may be publishable in some form, though as things stand, the current conclusions are not warranted by the data. Overall, the idea of speech rhythm and the experimental design are not particularly well informed by the literature. The experiments are based on a number of assumptions that lack direct support (especially the measurement of the regularity vs. irregularity condition), and the actual intelligibility of the stimuli is not adequately measured for all stimuli. I elaborate on the critical points below and offer some suggestions.

We thank the Reviewer for these critical points. We have endeavoured to address all these in this rebuttal letter and our revised manuscript. However, we respectfully disagree with the substantive criticisms expressed in the summary above which we believe misrepresent our work and our claims. For example, we do not claim *"that perception of certain timing regularities […] is only accurate"*, but rather that it is *more accurate* for intelligible than unintelligible speech. We are confident in this finding – the present manuscript offers several replications while ruling out all acoustic confounds. No form of acoustic measurement can explain our Experiment 3 finding that rhythm perception improves when acoustically identical stimuli are heard as intelligible. While our measurements of intelligibility are unsophisticated compared to published studies assessing the intelligibility of vocoded and sine-wave speech, they suffice to show that our manipulations changed intelligibility as intended. We thus disagree with the reviewer's assertion that "*current conclusions are not warranted by the data*".

Below we reply to each of those points individually. All page numbers refer to the version with tracked changes.

Major comments

(1) One major issue of the manuscript is the lack of an informed discussion and a clear working definition of the much-debated notion of speech rhythm, though it is in focus of the study. The

introduction describes the authors' own research and lacks a broader embedding in the relevant literature on the topic. There is an assumption that rhythm is necessarily isochronous (e.g. line 183 refers to "perfectly rhythmic speech"), though the generalisation from isolated words produced in time with a metronome to "speech rhythm" across the board is certainly a quantum leap of faith in terms of what "speech rhythm" might be. In line 752, there is a change of gears to "acoustic rhythms". What is it, exactly?

In our original submission, we had already clarified *"that our study was not designed to answer the long-standing debate on the importance of rhythm for the perception of speech [4–6,12–14]".* Neither did we claim that natural speech rhythm is isochronous as the Reviewer seems to imply, nor generalise our findings to natural speech.

We believe that this is the critical difference to previous studies on speech rhythm perception: we did not aim to test, manipulate or examine perception of *natural* speech rhythm. Rather, we tested how certain (acoustic and linguistic) manipulations of a rhythmic auditory stimulus affect our ability to detect irregularities in that rhythm. The advantage of our stimuli (isolated words produced in time with a metronome beat) is precisely that they are ordinarily perceived as isochronous and hence that detecting departures from this regularity requires accurate perception of rhythm. Given this difference, we also refrained from giving an extensive overview of previous findings on natural speech rhythm perception.
However, we agree with the Reviewer that these points need to be clear throughout the manuscript which we thoroughly revised accordingly.

In the revised Introduction we explain (p. 5):
"In this study, we take an approach that differs from previous work on speech rhythm perception. We explicitly refrain from testing or manipulating speech rhythm perception* per se. *Rather, we test how certain acoustic and perceptual manipulations that change the intelligibility of perceptually-rhythmic speech also affect our ability to detect irregularities in that rhythm. Our rhythmic speech is not as complex as connected speech, but nonetheless our stimuli are derived from naturally-produced speech and ecological: when counting, or reciting the alphabet, speakers will produce similar sequences that listeners perceive as rhythmically-regular monosyllables. This detail is important as our speech stimulus is* already perceptually rhythmic *– as explained below, the well-defined (but, admittedly, less

complex) rhythm allows a straightforward quantification of how these manipulations affect rhythm perception."

Throughout the manuscript, we revised various paragraphs to be more precise in the description of our aims and approaches. We also avoided the debated term "speech rhythm" where possible (including the title which now is: Intelligibility improves perception of timing changes in speech). For example (p. 6):

"[…] we carried out three experiments to further investigate how the linguistic properties of rhythmic speech affect the ability to detect violations in the stimulus rhythm […]."

The term "acoustic rhythms" is now more prominent in the manuscript as it comprises rhythms in unintelligible stimuli such as 1-channel vocoded speech.

To the general Discussion, we added (p. 35):

"[…] we show that making rhythmically-spoken speech intelligible improves the perception of this rhythm, but not that the rhythm of speech is important for its intelligibility."

(2) The original stimuli that served as the acoustic donors to the different types of acoustic degradation were produced in time with a metronome, leading to the assumption that the productions were indeed rhythmically regular (or rather, the timing of word onsets was isochronous). However, this was not checked. I am missing a test that would document the success of the intended production. In addition, there is a (not very well spelled out) assumption that speaking with a metronome leads to a tight alignment of p-centres of the stimuli with the metronome beats. This is likely to be the case, though I'd like to see this assumption substantiated by some kind of an acoustic measurement to demonstrate that in the regular condition, the acoustic pauses between the words were variable while the p-centre distances were kept relatively constant (and where exactly these p-centres were located). An overview of mean pause duration and mean inter-p-centre intervals (plus SDs or some measure of variability) is needed for the regular/irregular conditions. It might be helpful to get a linguist/phonetician on board, to help with sorting this out in an informed way. There is a substantial amount of literature out there (not referred to in the paper) showing that p-centre location falls close to the vowel onset, often coinciding with the moment of a fast spectral change. The authors claim acoustics do not play a role in the perception of speech rhythm (lines 800 – 806) but no acoustic measurements are actually taken.

It is quite possible that the best way forward for the manuscript is to discuss the results in terms of the perception of the p-centre location in degraded speech (instead of "speech rhythm"). Linguistic p-centres stir up acoustic isochrony, making the acoustics of language obey other principles than non-speech sounds and there is quite a bit of previous research (not reviewed in the paper) that shows this. I think the manuscript can make an important contribution to this research in that it shows how the p-centre effect disappears as speech signal gets unintelligible. For this framing to work, an experiment comparing natural vs. 16-channel noise vocoded productions would be needed though, to show that 16-channel noise vocoding behaves in the exact same way as natural speech (if it does). I am also missing the control condition – an acoustically regular version of the stimuli with equal duration of the pauses. It should be judged as regular in all stimuli that do not sounds like speech.

We believe that this comment is partly motivated by the misunderstanding that our study aims to contribute to the identification of "speech rhythm" in a spoken speech signal. As we tried to clarify above and in the revised manuscript, this is not the case.
In our case, the p-centre is the part of the monosyllabic words that the speaker aligned to the metronome beat. This should lead to p-centres in our stimulus sequences being isochronously spaced, although some degree of error in speech production cannot be ruled out. Importantly however, it is very unlikely that imperfectly spoken stimuli (i.e. not perfectly aligned to the isochronous metronome beat) can explain our results. Any perceptible imperfections would lead participants to reject our (intended) isochronous sequences as irregular. This would reduce detection of irregularities overall, without producing any of the observed differences between experimental conditions. We therefore believe that results do not necessarily need to be re-framed and analysed or discussed in terms of p-centre location. We hope that the Reviewer agrees with us. As explained in our response to the first point, the use of rhythmically spoken speech served to test effects of various manipulations on the participants' ability to detect rhythmic irregularities, but not to reveal corresponding effects on p-centre perception. We clarified this in our revised manuscript (p. 6):

"As in our previous study, we constructed sequences of five one-syllable words, spoken based on a metronome beat. We defined the p-centre of these words as that part of the word that the speaker (author MHD) aligned to the metronome beat. We assume that this led to p-centres in our five-word sequences being isochronously spaced, and therefore rhythmic in

perceptual terms. We then introduced irregularities in these rhythmic sequences by shifting one of the words towards another, and asked participants to decide whether they detected a rhythmic irregularity ("irregular trial") or not ("regular trial"). Although some imperfection during the metronome recording cannot be ruled out, imperfectly spoken stimuli (i.e. not perfectly aligned to the isochronous metronome beat) would only reduce detection of irregularities overall, without biasing specific experimental conditions."

We also added to the Discussion (p. 37):
"Other future work could examine whether and how the location of p-centres changes when speech becomes unintelligible, and how our results relate to the perception of rhythm in natural speech."

As for our claim that "acoustics do not play a role in the perception of speech rhythm", we believe that the Reviewer overlooked the double negation in the respective sentence: *"This result does, of course, not imply that acoustic properties – such as amplitude modulations or spectral detail – do not contribute to speech rhythm perception".* We emphasized this by making the first "not" italic.

(3) I don't see much value in Experiment-1 with regards to the overarching research goal of the manuscript. The intelligibility of the stimuli was not tested here, and I doubt that 1-channel vocoding sounded like speech at all. Looking at Figure 1, I expect the 1-channel vocoded manipulation to sound as some (slightly filtered) noise. In noise, there is no reason to expect a p-centre effect. Rather, one would expect an acoustic pause duration effect (i.e. sequencies with more equal durations of intervening acoustic silences would be judged as more "rhythmic" I assume they sounded just like noise – in this case, the conclusion that the authors are making is not warranted: It is not the isochrony that listeners stop being able to judge in these stimuli, it is the p-centre that is no longer a structuring unit of perception. In a way, the manuscript would be strengthened if Experiment-1 is removed and replaced by an experiment comparing natural and noise-vocoded productions.

As explained above, our study was not designed to examine how p-centre perception changes with our acoustic and linguistic manipulations. We observed that irregularities in 1-channel noise-vocoded speech are more difficult to detect than in 16-channel speech and reported the effect as such. The purpose of Experiment 1 was to replicate a previous

experiment with a higher proportion of irregular trials and a forced-choice paradigm. We believe that this successful replication will be interesting for some readers and sets the scene for later experiments in which we use similar methods to show that the perception of our rhythmic sequences is modified by intelligibility even when acoustic differences are controlled.

(4) Overall, the intelligibility of the stimuli is an issue. It is lacking in Experiment-1. Experiment-2 states that some noise-vocoded and spectrally rotated stimuli were perceived as "sometimes intelligible" by the listeners – can the authors be sure that this means listeners understood what was said? I have worked with spectrally rotated stimuli and don't quite believe this. Perhaps this rather means "sometimes sounding similar to speech" (in contrast to noise that cannot be interpreted as speech at all). As we know there can be substantial differences between self-perception and the actual behaviour, I think an additional test might be needed to check how many words can indeed be correctly recognised in the acoustic signals of different degradation levels. In Experiment-3, it is assumed that listeners can only learn to understand sinewave speech upon training or exposure, though we know some listeners recognise such sounds as speech without even much priming (e.g. Rosner et al. 2003, JSHLR).

In Experiments 1 and 2, we test the effect of spectral complexity and spectral rotation on performance in the irregularity detection task. A large number of previous studies – beginning with Shannon et al (1995, Science) have previously shown that 1-channel speech is unintelligible; vocoded speech with 16 channels approaches ceiling levels of intelligibility as measured by word report and subjective ratings (e.g. Davis & Johnsrude, 2003; Obleser, Eisner, & Kotz, 2008; Sheldon, Pichora, Fuller, & Schneider, 2008a; Sohoglu & Davis, 2016). For 16-channel rotated vocoded speech, previous studies are unanimous in confirming that this is entirely unintelligible – e.g. Scott et al, 2000; Narain et al, 2003; Lin et al, 2021. We confirmed all these observations using word report and post-hoc ratings already reported in the manuscript (Experiment 2, p. 20/21, Figure 3B). Given that the stimuli used in Experiment 1 are a subset of those that were assessed in Experiment 2 we are not persuaded that additional testing of the intelligibility of vocoded speech could add to the extensive existing literature or would modify the results or conclusions of our study.

In Experiment 3, we test how rhythm perception performance changes after training in sine-wave speech perception. In principle, these questions can be answered without any quantification of intelligibility. Nevertheless, we agree with the Reviewer that intelligibility is an important variable, and this is why we included a number of additional questionnaires/tests in the experiment to assess this. Although some of these tests indeed measure participants' subjective experience, we had already reported results from an additional, more objective test the Reviewer suggested (used in both Experiment 2 and 3; see, for example, Figure 3B and 5). This is described as the "word report task" (copied from p. 18): ***"Participants listened to one more example sound per condition […]. For each sound, they were told that the first word is always "pick" and the last word is always "up" and asked to write the three words in the middle. They were encouraged to guess if they were not sure."***

We did not claim that sine-wave speech can only be understood upon training or exposure as the Reviewer suggests. Nevertheless, we addressed the Reviewer's concern by adding the following:

Results (p. 30):
"Although a relatively large proportion of participants spontaneously described the stimulus as speech, this proportion increased after training by 22.0 %, 32.2 %, and 57.5 % in Groups 1, 2, and 3, respectively (based on responses immediately after vs immediately before training, see Procedure)."

Discussion (p. 34):
"Given the relatively large proportion of participants that spontaneously identified this stimulus as speech, it is likely that the training enhanced speech perception rather than changing it categorically. Nonetheless, the effect of training was to substantially modify speech intelligibility without changing the acoustic form of our sine-wave speech stimuli – which achieves our empirical goals."

(5) I notice that were quite a lot of participants excluded from the analyses, due to various reasons. Arguably, this is not the most methodologically rigorous way of doing stats. I wonder if a different approach would have some merit, e.g. an analysis based on non-aggregated data of regular/irregular responses using a glmer-model? This approach would have many

advantages, e.g. individual differences in the understanding of sine-wave speech without much practice can be estimated in Experiment-3 (some people naturally perceive such sounds as speech without having been primed first) and the direction of timing change (delayed/sped-up presentation of target words) can be estimated in Experiment-2. This way, meeting a threshold criterion is no longer a valid reason for excluding participants' data from the analyses. Additionally, the data can be modelled in a more sophisticated way, by including individually variable factors such as intelligibility and potentially acoustic factors of the stimuli.

We only excluded participants that: (1) were unable to do the task (chance-level performance in multiple conditions), (2) did not wear headphones in the online experiment (contra- to our specific instructions), or (3) showed signs of excessive fatigue as shown by strongly decreased performance in later task blocks (in particular for Experiment 3 that relied on performance differences between blocks). None of these criteria will bias our results; on the contrary, they might have reduced data quality and obscured otherwise informative outcomes. We verified (lines 590-592) that these exclusions did not modify results.

Minor comments

Lines 474 – 476: check your reference here, it does not seem to be correct, neither of the two studies supports the claim made

The corresponding sentence ("*it has been proposed that the amplitude envelope of certain key frequencies (around 1 kHz) that include vowel formants is the best predictor of the location of rhythmic beats in speech*") is supported by the acoustic measure of p-centre location given by Cummins & Port (1998), already cited in the original version of the manuscript. We removed the reference to the other study (Scott, 1998).

Line 511: a reference is needed here

In this sentence we refer to the possibility of task performance improving with practice. This is such a long established behavioural observation (going back to the Ancient Greeks, at least) that we felt no specific reference is needed.

Line 515: something is wrong with the sentence structure here.

We have made this sentence easier to read by adding additional punctuation:
"This design requires a larger number of participants than previously since: (1) the critical comparison of naïve and trained performance can only be performed in one order, and (2) each participant can only be tested once (since training effects are long-lasting)."

Lines 653-654: state the exact number of participants with chance-level d' who were excluded from the analyses

This number was already included in the "Participants" section, but we copied the information as requested (3, 2 and 4 in the three groups).

Lines 844-901: pure speculation without any obvious relationship to the results

In line with the Reviewer's comment, this section already includes the statement: ***"These explanations remain speculative and need to be explored in future studies."***

I suggest that the authors make (some of) their stimuli available as part of the submission (or in a repository like osf), for a better appreciation of the experimental designs and the conclusions.

This was already available for our original submission: ***"Example sounds and MATLAB code used for stimulus construction are available for all experiments (https://osf.io/p2ch8/)."*** **For our revised submission, we included anonymized data from individual subjects in the repository.**
