# Supplementary material for: Intelligibility improves perception of timing changes in speech
Source: PLoS One. 2023 Jan 12;18(1):e0279024. doi: 10.1371/journal.pone.0279024 (PMC9836318; doi:10.1371/journal.pone.0279024)
Supplement: Attachment — Submitted filename: shift_detection_REBUTTAL.docx [file pone.0279024.s002.docx]

**We thank the Editor and both Reviewers once more for their helpful comments that allowed us to improve our submission. We revised our manuscript according to these comments and provide our responses below. We have also made all stimuli available (**[**https://osf.io/p2ch8/**](https://osf.io/p2ch8/)**).**

**Reviewer #1:**

Many thanks to the authors for this thoughtful and thorough revision. They have addressed all of my questions, and I think the revisions in general have substantially improved the manuscript.

I have a few very minor comments, as follows:

Figure 1 caption (line 201): “Stimuli used in the Experiments 1 and 2” => “Stimuli used in Experiments 1 and 2”.

Lines 391 and 630: Thank you for including the data from the pseudo-audiogram procedure. However, I wonder if it might be better to call the cited figures a “threshold” rather than a “range”, since you’re only concerned with one end of the scale (i.e., the quietest sound detectable).

Line 770: “…only in the group that is trained relatively early.” For clarity, please could you specify which group you are referring to?

Lines 832-833: “…participants who perceive sine-wave speech as ‘speech’ (i.e. more intelligible)…” The parenthesised addition notwithstanding, I still feel as though this gives too much of an impression of a category shift from “non-speech” to “speech”. I’d therefore be inclined to soften the claim even further, perhaps describing the perception as more ‘speech-like’ (or something similar).

Line 833: “increased sensitivity to detect deviations” => “increased sensitivity to deviations”

Lines 895-896: “rhythmic patterns between language (e.g., stress-timed vs. syllable-timed [4])…” I appreciate the authors’ incorporation of a comment regarding cross-linguistic differences in speech rhythm. However, I feel I should point out that the “rhythm class” hypothesis they cite is now considered rather dated and controversial (see e.g., White et al, 2012; Nolan & Jeon, 2014). I think it’s perfectly fine to point to differences between languages, but I’d use a different reference if possible and avoid mentioning “stress-timed” and “syllable-timed” languages.

*White, L., Mattys, S. L., & Wiget, L. (2012). Language categorization by adults is based on sensitivity to durational cues, not rhythm class. Journal of Memory and Language, 66(4), 665-679.
*Nolan, F., & Jeon, H. S. (2014). Speech rhythm: a metaphor?. Philosophical Transactions of the Royal Society B: Biological Sciences, 369(1658), 20130396.

Once again, many thanks to the authors for this thought-provoking paper, which I very much enjoyed having the opportunity to read again.

**We are glad that we were able to address all questions and thank the Reviewer once again for constructive and detailed comments that helped us improve our manuscript. We have incorporated all of the suggestions above.**

**Reviewer #2:**

The manuscript is much improved, and accompanying clarifications provided by the authors are also very helpful indeed. Re: comment (1) - The terms and the assumptions of the study are now better spelled out, thank you. Re: my comment (2) - I appreciate the reluctance of engaging with the idea of a p-centre in-depth. My issue remains unaddressed though. I am not misunderstanding something, the calculation of the d-prime measure that quantifies participants' perceptions relies on p-centres being regular (hit) or irregular (miss). This (ir)regularity is an assumption that is not documented anywhere in the stimuli. The participants might be perceiving the timing of the unintelligible stimuli extremely well, better than the timing of intelligible stimuli even, but because timing is a hit/miss preconception that is not quantified in any way, the authors are bound to arrive at the conclusion that intelligibility improves perception of timing regularities. What if the timing is irregular in all of the unintelligible stimuli (i.e. under both hit and miss assumptions)? Re: comment (3) - I understand the rationale. Re: comment (4) - the issue has been partially addressed (does 1-channel noise-vocoded "speech" sound as speech at all?). Re: comment (5) - it was a recommendation, not a major issue.

**We are glad that we were able to address most of the issues the Reviewer raised and are grateful for their help in improving our manuscript further. In the following, all line numbers refer to the version with tracked changes.**

**(2) We appreciate the Reviewer’s concern and added further clarifications to our revised manuscript. Specifically, in line 143 we now emphasise that *“our definition of ‘irregular’ is entirely based on this temporal shift [shifting one of the words towards another] rather than p-centre positions of individual words.”* It is possible that noise-vocoding alters p-centre locations and this was already acknowledged in our original submission (line 882):**

**“*If amplitude fluctuations in this frequency band [1-channel speech] are distorted during the construction of these stimuli, this might lead to similarly distorted p-centres of individual words (i.e. they might not be equally spaced in time anymore), and thus to an erroneous perception of rhythm*.” This possibility, however, does not contradict our conclusion that the *same temporal shift* is easier to detect if noise-vocoded speech is intelligible than when it is not. Rather, it offers an explanation of why this could be (at least in Experiments 1 and 2): Distorted p-centres might hamper detection of this irregularity, even when broadband amplitude modulations and spectral detail are controlled for.**

**Finally, no form of acoustic differences can explain our Experiment 3 finding that rhythm perception improves when acoustically identical stimuli are heard as intelligible.**

**(4) We added the following details (line 92): *“16-channel noise-vocoded speech is clearly intelligible, even for naïve listeners, whereas 1-channel noise-vocoded speech sounds like noise to naïve listeners and is completely unintelligible when presented in isolation. 1-channel vocoded speech yields <5% word report accuracy when presented alone, but ~30 % when combined with visual cues like lip movements [22], suggesting that noise-vocoded speech can be perceived as speech-like, even when unintelligible.”***

**[22]** **Aller M, Økland HS, MacGregor LJ, Blank H, Davis MH. Differential Auditory and Visual Phase-Locking Are Observed during Audio-Visual Benefit and Silent Lip-Reading for Speech Perception. J Neurosci. 2022;42: 6108–6120.**
